# Supplementary material for: Complexome profiling of the Chlamydomonas psb28 mutant reveals TEF5 as an early PSII assembly factor
Source: Plant Cell. 2025 Mar 17;37(6):koaf055. doi: 10.1093/plcell/koaf055 (PMC12164590; doi:10.1093/plcell/koaf055)
Supplement: koaf055_Supplementary_Data [file koaf055_supplementary_data.zip › psb28_interactive.zip!/plots/intensity/qualityPlots/peptides/_Cre16.g684150.t1.2.html]

### Cre16.g684150.t1.2

Trivial name:   
SeqCoverage [median]: 12.50  
Mass: 28.43 kDa  
  
 

| Sequence | MissedCleavage | Mass [Da] | wtIntensity | muIntensity | Score |
| --- | --- | --- | --- | --- | --- |
| AIGEYAAR | 0 | 849.43 | 1979.49 | 10850.89 | 138.37 |
| AKDLAAQDVGAAR | 1 | 1284.68 | 1964.77 | 10752.05 | 85.54 |
| DLAAQDVGAAR | 0 | 1085.55 | 0.00 | 1668.36 | 82.75 |
|  |  |  | 3944.3 | 23271.3 |  |
